# AsKC11, a Kunitz Peptide from *Anemonia sulcata*, Is a Novel Activator of G Protein-Coupled Inward-Rectifier Potassium Channels

**DOI:** 10.3390/md20020140

**Published:** 2022-02-15

**Authors:** Dongchen An, Ernesto Lopes Pinheiro-Junior, László Béress, Irina Gladkikh, Elena Leychenko, Eivind A. B. Undheim, Steve Peigneur, Jan Tytgat

**Affiliations:** 1 Toxicology and Pharmacology, KU Leuven, Campus Gasthuisberg, O & N2, Herestraat 49, P.O. Box 922, 3000 Leuven, Belgium; dongchen.an@kuleuven.be (D.A.); ernesto.lopes@kuleuven.be (E.L.P.-J.); 2 Department of Internal Medicine, Division Experimental and Clinical Peptide Research, Pharis Biotech GmbH/Medical School Hannover, Feodor-Lynen-Str. 31, 30625 Hannover, Germany; dr.beress@t-online.de; 3 G.B. Elyakov Pacific Institute of Bioorganic Chemistry, Far Eastern Branch, Russian Academy of Sciences, 690022 Vladivostok, Russia; irinagladkikh@gmail.com (I.G.); leychenko@gmail.com (E.L.); 4 Centre for Biodiversity Dynamics, Department of Biology, Norwegian University of Science and Technology, N-7491 Trondheim, Norway; e.a.b.undheim@ibv.uio.no; 5 Centre for Ecological and Evolutionary Synthesis, Department of Bioscience, University of Oslo, N-0316 Oslo, Norway; 6 Institute for Molecular Bioscience, The University of Queensland, Brisbane, QLD 4072, Australia; 7 Centre for Advanced Imaging, The University of Queensland, Brisbane, QLD 4072, Australia

**Keywords:** sea anemone venom, AsKC11, GIRK1/2, potassium channels, brain diseases

## Abstract

(1) Background: G protein-coupled inward-rectifier potassium (GIRK) channels, especially neuronal GIRK1/2 channels, have been the focus of intense research interest for developing drugs against brain diseases. In this context, venom peptides that selectively activate GIRK channels can be seen as a new source for drug development. Here, we report on the identification and electrophysiological characterization of a novel activator of GIRK1/2 channels, AsKC11, found in the venom of the sea anemone *Anemonia sulcata*. (2) Methods: AsKC11 was purified from the sea anemone venom by reverse-phase chromatography and the sequence was identified by mass spectrometry. Using the two-electrode voltage-clamp technique, the activity of AsKC11 on GIRK1/2 channels was studied and its selectivity for other potassium channels was investigated. (3) Results: AsKC11, a Kunitz peptide found in the venom of *A. sulcata*, is the first peptide shown to directly activate neuronal GIRK1/2 channels independent from Gi/o protein activity, without affecting the inward-rectifier potassium channel (IRK1) and with only a minor effect on K_V_1.6 channels. Thus, AsKC11 is a novel activator of GIRK channels resulting in larger K^+^ currents because of an increased chord conductance. (4) Conclusions: These discoveries provide new insights into a novel class of GIRK activators.

## 1. Introduction

G protein-coupled inward-rectifier potassium (GIRK/Kir3) channels are members of a large family of inward-rectifying potassium (Kir1-7) channels, which have been studied intensively for nearly three decades [1,2,3]. GIRK channels exist as tetrameric complexes comprising either identical (homomeric) or similar (heteromeric) subunits that encode a K^+^ selectivity-filter-containing transmembrane pore (TMD for transmembrane domain) serving to conduct potassium ions and a cytoplasmic domain (CTD) [3,4]. The TMD and CTD are covalently linked by a tether, called the TMD-CTD linker [4]. GIRK channels are important regulators of the cellular excitability in the brain and cardiac cells, maintaining the resting membrane potential and regulating the shape and duration of the action potential in excitable cells [5]. In native tissues, GIRK channels are activated by binding of the βγ subunit of Gi/o proteins (Gi/o(βγ)) which disassociates from the Gi/o(α) subunit following the activation of PTX-sensitive G protein-coupled receptors (GPCRs) [6,7]. Thus, the activation of GIRK channels by GPCRs is a crucial part of signal transduction evoked by a diversity of GPCR agonists, including endogenous neurotransmitters such as acetylcholine, dopamine, opioids, serotonin, somatostatin, adenosine, and GABA, as well as exogenous molecules, such as WIN55,212-2 and CP55,940, which are the agonists of cannabinoid receptors [1,2,7,8]. 

The mammalian GIRK family is composed of four domains (GIRK1, GIRK2, GIRK3, and GIRK4 channels) encoded by KCNJ3, KCNJ6, KCNJ9, and KCNJ5 genes, respectively [9]. Among them, GIRK1, GIRK2, and GIRK3 channels are expressed in the brain and form either homotetramers of GIRK2 channel or heterotetramers (e.g., GIRK1/2 channels) in various brain regions with a high presence in the olfactory bulb, hippocampus, cortex, thalamus, and cerebellum, as well as in the spinal cord [1,5]. The prototypical functional form of GIRK channels in the brain consists primarily of heterotetrameric GIRK1/2 channels [5,9]. They control neuronal excitability and neurotransmission, confer bistability (transition from asynchronous spikes to synchronous bursts of activity) to neural networks [1]. The wide distribution of GIRK channel subunits in the brain, and their function to regulate neuronal excitability by mediating many actions of major neurotransmitters, suggest important physiological roles for GIRK channels in the central nervous system, e.g., general homeostasis, particular synaptic plasticity processes, learning, memory, pain signaling, and mood control [1,8,10]. Moreover, several studies have shown the functions of GIRK channels in pathological conditions. Pharmacologically increasing the activity of GIRK channels can induce efficacy in rodent models of numerous neurological or psychiatric disorders, for instance, antiseizure, anxiolytic, antinociceptive, and rescue of amyloid-β-evoked deficits in hippocampal function [2,3,11,12,13,14,15,16,17,18]. These studies suggest the therapeutic potential of activating neuronal GIRK channels, especially the primary GIRK1/2, to treat brain diseases in humans [5]. 

To better understand the potential of activating neuronal GIRK1/2 channels for therapeutic intervention, selective and potent activators have been developed for GIRK channels. A synthetic small molecule, ML297, which is asymmetrical urea and is the first selective and potent activator of GIRK1/2 channels, stands as the prototype among a series of discovered small molecules, exhibiting an affinity of activity in the nanomolar range [2,5,16]. The property of activating GIRK channels of ML297 is closely relevant to its antiepileptic and anxiolytic effects in animal models [2,16]. However, the suboptimal pharmacokinetic properties of ML297 have hindered its further clinical application [2]. Thus, the continued work has been focused on improving druglike properties of selective activators for neuronal GIRK1/2 channels, leading to the most recent discovery of ML297 analogs, GAT1508, GiGA1, and VU0810464 [15,17,18]. Two critical GIRK1 residues, Phe-137 and Asp-173, are necessary and sufficient for the activation of GIRK1/2 channels mediated by ML297 [16]. Like ML297, the GAT1508-binding subunit GIRK1 also utilizes these two key residues (Phe-137 and Asp-173) to bind and transduce the activation effect of this small molecule activator [18]. In contrast, GiGA1 targets the alcohol pocket where Leu246 in GIRK1 and Leu257 in GIRK2 is essential for the activation effect of GiGA1 [15].

In the present work, we have looked at animal venoms as a source for novel GIRK channel ligands. Venoms are known as a tremendous treasure-house of peptide toxins (i.e., venom peptides) that specifically, potently, stably, and speedily manipulate physiological targets such as ion channels, including GIRK channels and receptors [19]. Thus, peptides found in the venom of different animals are providing tools to investigate and modulate these macromolecular targets [20]. Moreover, they are reliable alternatives for small nonpeptidic compounds for drug development, owing to the higher selectivity [19,21]. In addition, peptides may be metabolized and cleared without accumulation in body tissues, thereby minimizing the occurrence of side effects [21]. As such, the venom peptide activator of GIRK channels is an interesting template for further drug design.

Sea anemones (phylum Cnidaria, class Anthozoa) produce various classes of peptide toxins that target a diverse array of ion channels/receptors, including Kunitz-type peptide toxins [22]. Some of these Kunitz peptide toxins, such as ShPI-1, HCRG21, and AsKC1, are modulators acting on different voltage-gated potassium (K_V_) and transient receptor potential cation channel subfamily V member 1 (TRPV1) [23,24,25]. In the sequence of these peptide toxins, the Kunitz motif is a cysteine-rich peptide chain of ~ 60 amino acid residues with an alpha and beta fold, stabilized by three conserved disulfide bridges [26]. Kunitz-type peptides are not only found in sea anemones, but also snakes, spiders, scorpions, and cone snails [27]. They show diverse biological activities, such as inhibition of proteases and/or blocking or modulating ion channels [26]. For instance, some Kunitz-type peptides from venomous animals modulate type 2 vasopressin receptors and integrins [28,29] or TRPV1 [30], K_V_ channels [23,25,31,32,33], voltage-gated sodium (Na_V_) channels [34], voltage-gated calcium (Ca_V_) channels [35], and acid-sensing ion channels (ASIC) [36]. None of the peptide toxins were shown to be an activator of GIRK channels.

In search of novel ligands that might have the potential for developing activators of GIRK1/2 channels to treat brain diseases, we screened venoms of sea anemones to find a novel activator for GIRK1/2 channels. Here, for the first time, we report the purification, identification, and electrophysiological characterization of the venom peptide of sea anemone *Anemonia sulcata*, called AsKC11. This Kunitz-type peptide is the first peptidic activator of GIRK1/2 channels. Interestingly, AsKC11 shows no effects on the inward-rectifier potassium channel 2.1 (Kir2.1 or IRK1) and exhibits low affinity for K_V_1.6 channels. The discovery of AsKC11 represents a significant advancement in our ability to uncover a new class of activators of GIRK channels as well as providing a novel basis to find useful tools for fully probing the role and therapeutic potential of GIRK channels.

## 2. Results

### 2.1. Enhancement of K^+^ Currents through GIRK1/2 Channels by the Venom Fraction from Anemonia Sulcata

**Figure 1:**
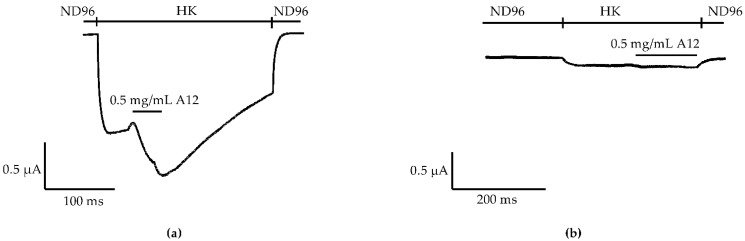
AsKC11 enhanced K^+^ currents in oocytes coexpressing GIRK1/2 but not in noninjected oocytes. (**a**) A representative current trace shows that the enhancement of K^+^ currents (I_K,A_) through GIRK1/2 channels was induced by the active venom fraction A12 in *Xenopus laevis* oocytes (*n* = 6). (**b**) A representative current trace shows that no appreciable currents were induced by the active venom fraction A12 in noninjected *Xenopus laevis* oocytes (*n* = 3).

To identify the novel activator of GIRK1/2 channels, we first tested the effects of venom fractions previously purified from the venom of *Anemonia sulcata* [25] on whole-cell currents in oocytes expressing GIRK1/2 channels. After screening, one of the venom fractions, named A12, was found to enhance the K^+^ currents carried by the GIRK1/2 channels in the oocytes. The oocytes coinjected with GIRK1/2 cRNA were voltage-clamped at −90 mV. To increase the GIRK current at the negative potential, a high-potassium extracellular buffer (HK) was used, which allowed for more obvious currents and accurate measurement. Thus, after exchanging ND96 to HK, basal K^+^ currents (I_K,basal_) were observed and were carried by GIRK1/2 channels (Figure 1a). In the presence of HK, K^+^ current enhancement was immediately evoked on the application of 0.5 mg/mL fraction A12 (I_K,A_) and was reversible following washing out of this fraction (Figure 1a). Since no appreciable current response to this fraction could be recorded in noninjected oocytes (Figure 1b), the increase in K^+^ currents in oocytes expressing GIRK1/2 channels can be attributed to the activation of GIRK1/2 channels.

### 2.2. Purification and Identification of AsKC11 from the Active Venom Fraction

**Figure 2:**
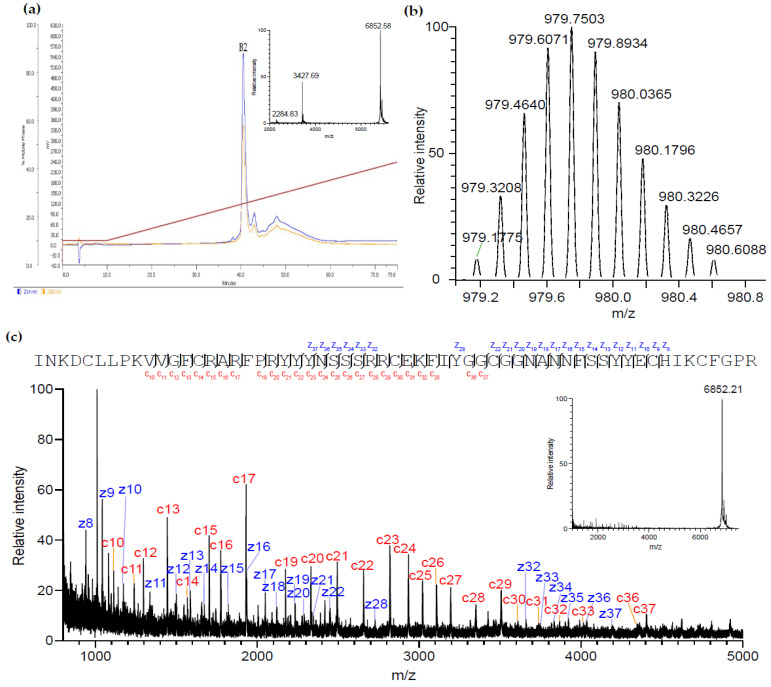
Purification and identification of the active peptide enhancing K^+^ currents through GIRK1/2 channels: (**a**) the RP-HPLC profile shows the subpurification of the venom fraction A12 which was previously purified from the venom of *Anemonia sulcata*, using Vydac C18 (4.6 mm, 5 μm, 25 cm) with a linear gradient of 10% to 90% acetonitrile (ACN) and 0.1% trifluoroacetic acid (TFA) over 175 min at a flow rate of 1 mL/min. UV absorptions were monitored at 214 nm (blue line) and 280 nm (yellow line). MALDI-TOF spectrum of the fraction containing B2 obtained in linear positive mode is shown as inset, with MH^+1^, MH^+2^, and MH^+3^ ions of B2 labeled. (**b**) Isotope envelope of B2 MH^+7^ measured on a Thermo Q Exactive mass spectrometer. (**c**) Identification of the amino acid sequence of the mature B2 by MALDI-ISD-MS. C- and z-ions are mapped onto the amino acid sequence of B2, while the corresponding peaks are labeled in a zoomed-in view across the main *m/z* region of interest. The full mass spectrum that was acquired for the MALDI-ISD-MS experiment is shown as an inset.

The fraction A12 showed the ability to enhance the K^+^ currents via GIRK1/2 channels and was further purified by reverse-phase high-performance liquid chromatography (RP-HPLC) (Figure 2a). This subpurification step allowed the separation of the major peptide B2, with an average mass of 6,851.6 Da. 

**Figure 3:**
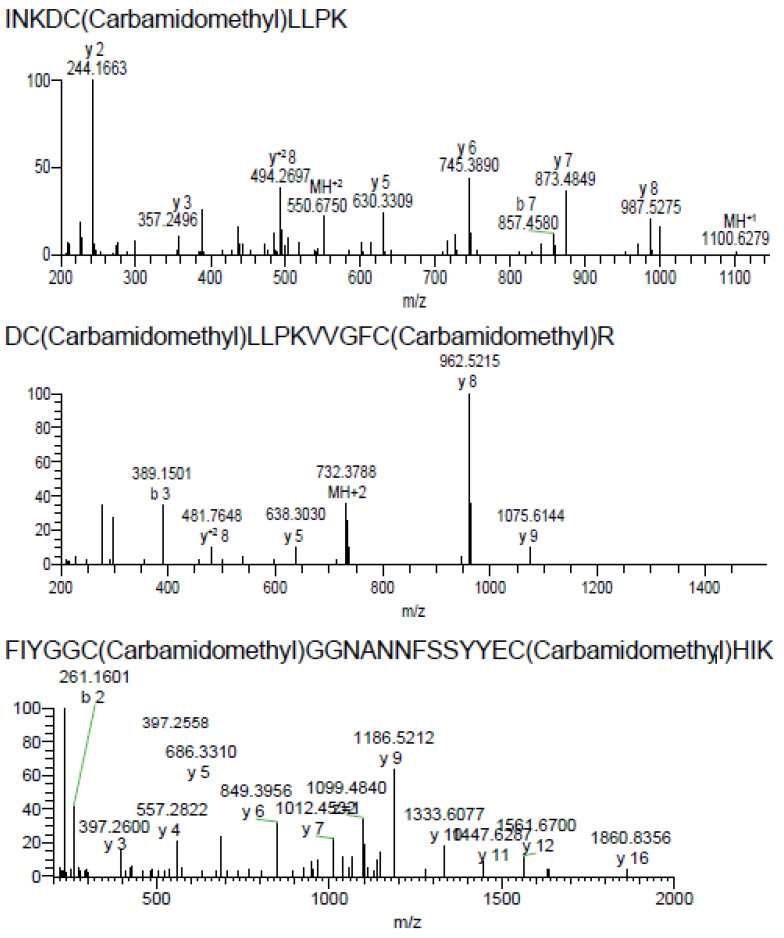
The amino acid sequence of the peptide B2 was validated by LC-MS/MS.

Searching LC-MS/MS spectra from reduced and alkylated and trypsin-digested B2 against a custom database consisting of 138,187 unique amino acid sequences, we identified two putative transcripts (NCBI accession codes FK727748 and FK749190) from *Anemonia viridis* encoding near-identical prepropeptides. Accurate mass measurement showed that the monoisotopic molecular mass of the native peptide was 6,847.22 Da (Figure 2b), while its sequence of 59 residues was determined by in-source decay during MALDI-TOF using a 1,5-diaminonaphthalene matrix (Figure 2c). To further validate the amino acid sequence of B2 we also manually examined the LC-MS/MS data, which yielded complete and high-confidence coverage of the sequence of the mature B2 determined by MALDI-ISD-MS (Figure 3).

**Figure 4:**
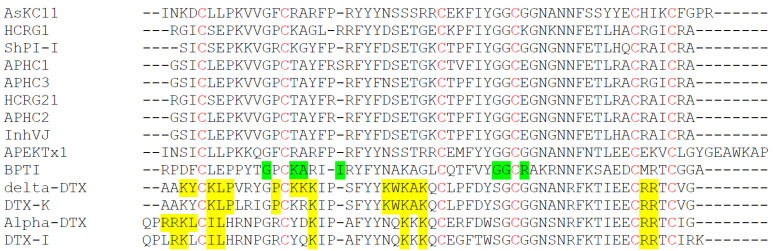
A multiple sequence alignment of AsKC11 and homologous Kunitz-type peptides. AsKC11: snakelocks anemone *Anemonia sulcata*, a synonym of *Anemonia viridis*, Uniprot accession number: P0DN15. HCRG1: leathery sea anemone *Heteractis crispa*, Uniprot accession number: C0HJU6. ShPI-I: sun anemone *Stichodactyla helianthus*, Uniprot accession number: P31713. APHC1: leathery sea anemone *Heteractis crispa*, Uniprot accession number: B2G331. APHC3: leathery sea anemone *Heteractis crispa*, Uniprot accession number: C0HJF3. HCRG21: leathery sea anemone *Heteractis crispa*, Uniprot accession number: P0DL86. APHC2: leathery sea anemone *Heteractis crispa*, Uniprot accession number: C0HJF4. InhVJ: leathery sea anemone *Heteractis crispa*, Uniprot accession number: P0DMJ5. APEKTx1: green aggregating anemone *Anthopleura elegantissima*, Uniprot accession number: P86862. BPTI: bovine *Bos taurus*, Uniprot accession number: P00974. Delta-DTX: eastern green mamba *Dendroaspis angusticeps*, Uniprot accession number: P00982. DTX-K: black mamba *Dendroaspis polylepis*, Uniprot accession number: P00981. Alpha-DTX: eastern green mamba *Dendroaspis angusticeps*, Uniprot accession number: P00980. DTX-I: black mamba *Dendroaspis polylepis*, Uniprot accession number: P00979. Cysteine residues are indicated in red, key residues for K_V_ channel inhibition are boxed in yellow, key residues for trypsin inhibition are boxed in green.

We next performed a standard protein Basic Local Alignment Search (BLAST, NCBI) using the blastp algorithm to identify hits. Among them, 33 hits were found in the UniprotKB Protein knowledgebase belonging to the Swiss-Prot section of UniProtKB. The Kunitz peptide U-actitoxin-Avd3n, also named AsKC11, found in *A. viridis,* which is a synonym of *A. sulcata,* was identified as sharing the same primary sequence of the peptide B2. Therefore, the peptide B2 was named AsKC11, belonging to the venom Kunitz-peptide family. The 20 hits showing more than 70% identity with AsKC11 are all from the *Anemonia* genus and most of them are from *A. viridis*. A multiple sequence alignment of AsKC11 with well-characterized venom Kunitz-type peptides and BPTI is shown in Figure 4.

To carry out more functional tests with more material, recombinant AsKC11 (rAsKC11) was produced. The yield of the rAsKC11 was about 330 µg from 1 L of the cellular culture. The recombinant peptide had the same retention time of 41 min by RP-HPLC, and when natural and recombinant peptides were coadministered, one HPLC peak was obtained. The molecular mass of rAsKC11 was 6,851.7 Da as the toxin B2. Additionally, it was found to increase the K^+^ currents through the GIRK1/2 channels in the presence of HK, like the native peptide. These data indicate that the Kunitz peptide AsKC11 in the fraction A12 from the venom of *A. sulcata* is the active peptide responsible for enhancing the K^+^ currents through GIRK1/2 channels. 

### 2.3. Potency of AsKC11 at Enhancing GIRK1/2 Channel-Carried Inward K^+^ Currents

**Figure 5:**
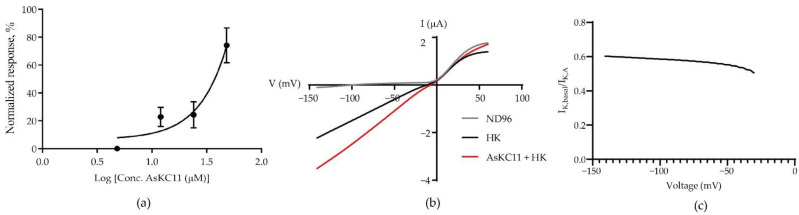
The concentration-dependent effect of AsKC11 on inward K^+^ currents through GIRK1/2 channels: (**a**) The concentration–response curve for AsKC11-evoked inward K^+^ currents. The amplitude of the I_K,A_ was normalized to the amplitude of the I_K,basal_. Data represent at least three independent experiments and are presented as mean ± SD. (**b**) A representative trace of AsKC11 (48 µM)-evoked enhancement of inward K^+^ currents through GIRK1/2 channels in oocytes. The measurement was conducted with the 1-s voltage ramp protocol from −150 to +60 mV from a holding potential of −20 mV. (**c**) The curve of I_K,basal_ divided by I_K,A_ (from −140 mV to −30 mV) shows the change in the inward rectification of GIRK1/2 channels in the presence of AsKC11 (48 µM). Each point represents the average value of I_K,basal_ divided by I_K,A_ of four independent experiments. SD of each point < 0.04, not shown in the figure.

The potency of AsKC11 to enhance inward K^+^ currents through GIRK1/2 channels was studied by applying increasing concentrations of AsKC11 (2.4, 12, 24, and 48 µM) to oocytes expressing GIRK1/2 channels. These oocytes were subjected to a 1-s voltage ramp protocol from −150 to +60 mV from a holding potential of −20 mV. The K^+^ current enhancement elicited by AsKC11 was concentration dependent, as shown in Figure 5a. 

Furthermore, according to the currents measured with the voltage ramp protocol, we could observe that the reversal potentials measured for basal and AsKC11-induced currents through GIRK1/2 channels were comparable and close to 0 mV (Figure 5b). Thus, AsKC11 did not affect the reversal potential of the measured currents, but simply increased its amplitude. This current enhancement was presumably a result of increased GIRK1/2 channel chord conductance. The impact on the inward rectification of GIRK1/2 channels was hardly observed in the presence of 48 µM AsKC11 (Figure 5c).

In addition to heterotetrameric GIRK1/2 channels, the GIRK2 channel can also form homomultimers in the human body. To further address the relationship between AsKC11 and GIRK1/2 channels, we investigated the effect of 48 μM AsKC11 on inward K^+^ currents through the homotetrameric GIRK2 channel. In oocytes expressing the GIRK2 channel, 48 μM AsKC11 enhanced K^+^ currents carried by the GIRK2 channel to approximately half the extent of amplitudes (43.7 ± 14.7% enhancement in inward K^+^ currents, *n* = 6) compared to heterotetrameric GIRK1/2 channels (74.1 ± 12.5% enhancement in inward K^+^ currents, *n* = 4) (data not shown).

### 2.4. AsKC11-Evoked Activation of GIRK1/2 Channels Independent from Gi/o

**Figure 6:**
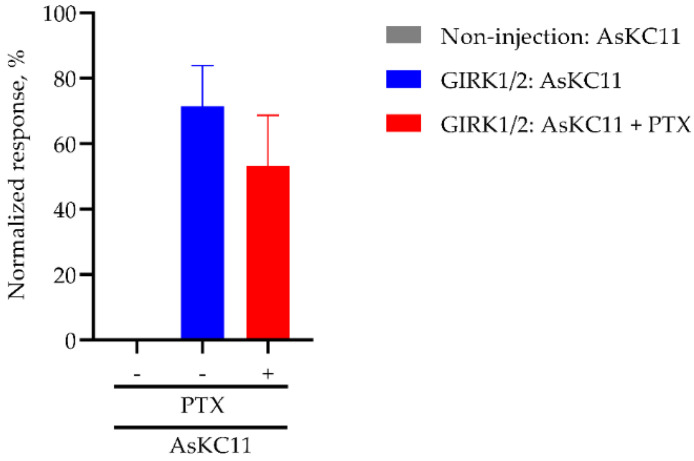
Summary of PTX effect on the response induced by AsKC11 (48 µM) in oocytes expressing GIRK1/2 channels. The amplitude of the I_K,A_ was normalized to the amplitude of the I_K,basal_. No significant difference in the current response between the experimental groups GIRK1/2: AsKC11 (*n* = 5) and GIRK1/2: AsKC11 + PTX (*n* = 3) (parametric unpaired *t*-test with Welch’s correction, *p* = 0.167). The gray column representing the control group Non-injection: AsKC11 is invisible since no appreciable current was induced by AsKC11 in noninjected oocytes (*n* = 3).

To assess whether AsKC11-induced GIRK channel activation requires Gi/o GPCRs, we investigated AsKC11-elicited K^+^ currents carried by GIRK1/2 channels in the absence and presence of pertussis toxin (PTX) which directly inactivates Gi/o by ADP ribosylation. First, 2.5 ng of PTX was injected into the oocytes expressing GIRK1/2 channels one hour before the measurement. These oocytes were then voltage-clamped at −90 mV for measuring. As shown in Figure 6, in oocytes expressing GIRK1/2 channels, PTX treatment did not affect the response amplitudes evoked by 48 μM AsKC11. Thus, unlike the activation of GIRK channels mediated by GPCRs through Gi/o, we found that the effects of AsKC11 are not affected by the PTX treatment. These data support the concept that AsKC11 acts directly at the GIRK1/2 channels and does not require the presence of an activated Gi/o GPCR. Therefore, AsKC11 could be considered as a novel activator of GIRK1/2 channels.

### 2.5. Affinity of AsKC11 to GIRK1/2 Channels

As shown in the abovementioned results, AsKC11 binding was reversible. We next explored whether the AsKC11-elicited activation of GIRK1/2 channels followed a kinetic behavior of a simple bimolecular reaction. The current activation upon AsKC11 application and recovery upon AsKC11 removal followed a single exponential time course compatible with a bimolecular reaction scheme (See Section 4). As required by the biomolecular scheme, for the 48 μM AsKC11-evoked activation of GIRK1/2 channels, the apparent first-order association rate constant (*k_on_*) was 5.71 × 10^−3^ s^−1^, and the first-order dissociation rate constant (*k_off_* = β) was 6.33 × 10^−3^ s^−1^. The second-order association rate constant (α) was 7.82 × 10^−5^ μM^−1^ s^−1^. When using these values to calculate the affinity of AsKC11 to GIRK1/2 channels, a *k_d_* value of 80.9 μM is found. Assuming the relationship between the channel occupancy and response is linear, *k_d_* equals EC_50_ = 80.9 μM.

### 2.6. Effect of AsKC11 on Different Potassium Ion Channels

**Figure 7:**
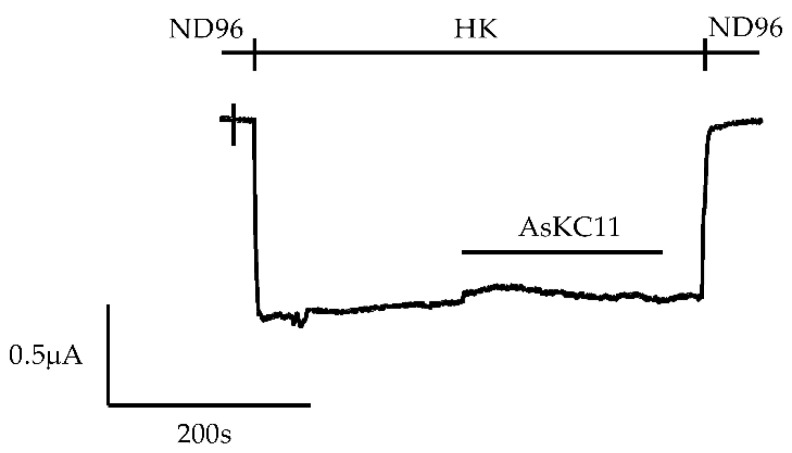
Selectivity screening on IRK1. No alteration of the current was observed after the addition of AsKC11 (48 μM) to IRK1 (*n* = 3).

To explore the selectivity of AsKC11 for GIRK1/2, we tested it on oocytes expressing the closely related potassium channel IRK1 as well as five voltage-gated potassium channels, K_V_1.1–1.4 and K_V_1.6. At a concentration of 48 μM, AsKC11 was inactive on IRK1 (*n* = 3) (Figure 7). 

**Figure 8:**
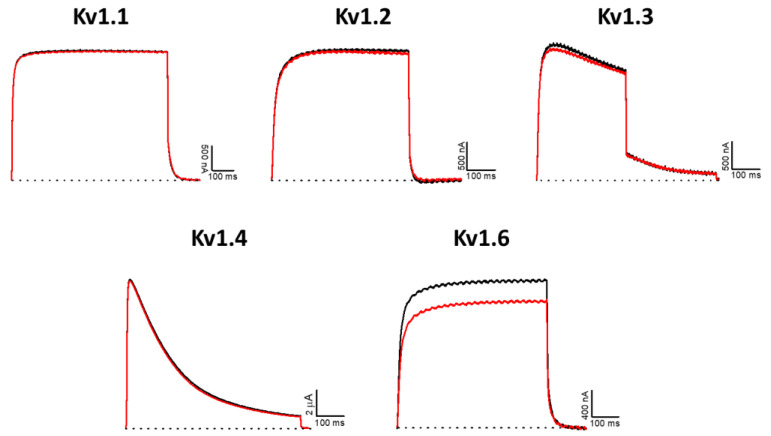
Electrophysiological characterization of AsKC11 (1 µM) on a panel of K_V_1 channels. The black lines represent the control condition, while the red lines indicate the current obtained after the addition of the peptide. Dotted lines represent 0 current levels. The graphs illustrate the effects obtained in a series of at least three independent experiments (*n* ≥ 3).

For K_V_1 channels, most marine toxins exert obvious effects with ~1 μM [22]. In this experiment, no significant effect was observed for K_V_1.1–1.4 channels, although a small inhibition was recorded for K_V_1.6 (13.5 ± 2.9%) (*n* ≥ 3) by applying 1 μM AsKC11 (Figure 8). 

## 3. Discussion

Currently, the activation of neuronal GIRK channels, specifically GIRK1/2, is considered a promising pharmaceutical strategy against many neurological and psychiatric conditions, such as epilepsy, hyperalgesia, and neurodegenerative diseases [1,3,5,6]. In this article, we report the identification of AsKC11, a Kunitz toxin/peptide found in *Anemonia sulcata*, that activates GIRK channels. Kunitz-scaffold toxins have also been found in the venoms of snakes, spiders, solitary wasps, scorpions, polychaete worms, cone snails, and gorgonian corals [37]. They show diverse biological activities such as inhibition of proteases and/or blocking or modulating ion channels [26]. AsKC11 is the first peptide-type activator of neuronal GIRK1/2 channels with a deduced EC_50_ of 80.9 µM. The other known peptide ligand of GIRK channels is a selective and potent GIRK inhibitor tertiapin (TPN), a peptide toxin found in the venom of honeybee *Apis mellifera* [38]. TPN inhibits the GIRK1/4 channels with nanomolar affinities [38]. Thereafter, the stable derivative of the bee venom toxin TPN was generated and was named TPNQ [39]. So far, TPNQ has been one of the most useful molecular probes for studying GIRK channels.

Sea anemones are rich in various bioactive neuropeptide toxins [40]. These peptide toxins have been applied to neuroscience research tools or directly developed as marine drugs [40]. Typically, neuropeptide toxins are among the main peptide/protein components of sea anemone venoms and act on Na_V_ channels, K_V_ channels, ASIC, and other ion channels [40,41]. AsKC11 is unique because it is a sea anemone Kunitz toxin active on neuronal GIRK1/2 channels. This peptide is a major component and its role as a part of the sea anemone venom has not been determined yet. 

In terms of the structure, AsKC11 belongs to K_V_ type 2 anemone toxins which all contain a Kunitz-type motif. New K_V_ type 2 anemone toxins are supposed to be protease inhibitors and K_V_ channel blockers, inferred from the sequence similarity according to the UniprotKB Protein knowledgebase and NCBI BLAST [22,42]. Thus, AsKC11 was assumed to inhibit K_V_1 channels at the beginning of the study. However, data of the experiments show that AsKC11 at 1 µM was inactive on K_V_1.1–1.4 channels, while the K_V_1.6 channel was mildly blocked by 1 µM AsKC11. Compared to the most marine toxins significantly blocking K_V_1 channels in a nanomolar range [22], AsKC11 shows much less potency on K_V_1 channels. This could be partly explained by the lack of sufficient key residues which are essential for the high affinity of Kunitz peptides to K_V_1 channels. According to the sequence homology shown in the alignment (Figure 4), only two positively charged amino acid residues (arginine and lysine) important for K_V_ channel activity are conserved in AsKC11 [23,43,44]. Moreover, AsKC11 most likely exerts protease inhibition activity since most of the major and minor protease binding sites are conserved, as shown in Figure 4 [23,43,45]. 

Previously, the only reported exogenous GIRK activators were small non-peptidic molecules, ethanol and the natural product—naringin—activating GIRK channels with very low potency, as well as some compounds of undisclosed structure [3,38,46]. These activators are weak and nonspecific since they exert effects in the millimolar range and target several types of ion channels at the same range of concentrations [3,46,47,48]. Thus, early investigation of the pathological and physiological relevance of GIRK channels was hampered partly by the lack of potent and selective probes [39]. Technologies that enable the screening of large libraries consisting of thousands of compounds boosted the discovery of many small molecules that specifically activate GIRK channels in the absence of an active Gi/o-coupled GPCR [3]. ML297, as the prototype of selective and potent GIRK activators, is now one of the most used pharmacological tools to probe GIRK functions. The activation of GIRK1/2 evoked by ML297 required the presence of the membrane phospholipid phosphatidylinositol 4,5-bisphosphate instead of Gi/o, therefore exhibiting a GPCR-independent mechanism of channel activation [2]. Like ML297, AsKC11 directly activates GIRK1/2 channels, independent from Gi/o. Additionally, AsKC11 significantly activated the homotetrameric GIRK2 channel. In contrast, ML297 has been reported to not affect the GIRK2 channel [2,16]. Scientists have been making efforts to ameliorate the pharmacokinetic profile of the small molecules specifically activating GIRK channels and improving their selectivity towards neuronal GIRK1/2. Though AsKC11 activated GIRK1/2 with low potency in the current study, it sheds light on a new horizon for discovering a novel class of GIRK activators.

## 4. Materials and Methods

### 4.1. AsKC11 Identification

The venom fractions were purified from the venom of *Anemonia sulcata* as previously described [25]. The fraction A12 was further purified and AsKC11 contained in this fraction was isolated using a C18 column (Vydac, 4.6 mm, 5 μm, 25 cm, flow rate = 1 mL/min) by RP-HPLC (Gilson, Villiers-le-Bel, France), using a linear gradient from 10% to 90% acetonitrile (ACN) and 0.1% trifluoroacetic acid (TFA) within 175 min. 

Approximately 5 µg AsKC11 was diluted in 8 µL 10% ACN 100 mM ammonium bicarbonate, pH 8, reduced with 5 mM dithiothreitol at 70 °C for 5 min, and alkylated with 10 mM iodoacetamide at 30 °C for 30 min. The reduced and alkylated AsKC11 was digested with trypsin (20 ng/µL in 10 µL) at 37 °C overnight and the resulting tryptic peptides were desalted using a C18 ZipTip (Thermo Fisher, USA), dried by vacuum centrifugation, and redissolved in 0.5% formic acid. Then, 1 µg of reduced, alkylated, and digested AsKC11 was analyzed by LC-MS/MS on a Q Exactive Fourier transform mass spectrometer (Thermo Fisher, USA) coupled to an Ultimate 3000 nano-UHPLC system (Dionex, USA). Peptides were separated on an Acclaim PepMap 100 column (C18, 3 µm particles, 100 Å pore size, 75 μm inner diameter) (Dionex, USA) using a flow rate of 300 nL/min and a gradient of 3–80% solvent B (90% ACN, 0.1% FA) in 0.1% FA over 27 min. Survey MS spectra were acquired across 400–2,000 *m/z* at a resolution of 70,000 at 200 *m/z*. Higher-energy collisional dissociation (HCD) MS2 scans were performed at 200–2,000 *m/z* at a resolution of 17,000 using an isolation width of 2.0 *m/z*, a threshold of 1e5 ion counts, and a maximum accumulation time of 60 ms. 

To measure the mass of AsKC11, 200 ng peptide was analyzed by LC-ESI-MS as above and by MALDI-TOF-MS using an UltraFlex II (Bruker) operated in positive linear mode with α-cyano-4-hydroxycinnamic acid (CHCA) (7 mg/mL in 50% ACN, 0.2% TFA) as the matrix. To obtain amino acid sequence information from the intact, native AsKC11 by MALDI-ISD-MS, we spotted 1 µL peptide (1 µg/µL in 1% formic acid 50 % ACN) with 1 µL saturated 1,5-diaminonaphthalene (1,5-DAN) solution in 100% methanol. Spectra were acquired from 800 to 7,000 *m/z* using the same Bruker UltraFlex II operated in linear positive mode using approximately 10% higher laser power than with CHCA. Fragment ions were used for both manual de novo sequencing and manually compared to theoretical fragment ion mass lists obtained using the Protein Prospector MS-Product tool (https://prospector.ucsf.edu/prospector/cgi-bin/msform.cgi?form=msproduct, accessed on 10 January 2022).

### 4.2. Recombinant Synthesis of rAsKC11

The construction pET32b(+)/AsKC11 was synthesized by JSC Eurogen (Russia). The recombinant plasmid with target genes was used for the transformation of BL21(DE3) *Escherichia coli* cells by electroporation. The transformed cells were cultured in LB medium (600 mL) containing carbenicillin (100 µg/mL) at 37 °C to the optical density of A600 0.6–0.8. Isopropyl-β,D-thiogalactopyranoside (IPTG) was added to the final concentration of 0.3 mM for the expression induction. The cells were grown for 17 h at 18 °C. The bacterial cells were precipitated from the solution by centrifugation at 6,000 rpm for 7 min.

The fusion protein that contained thioredoxin and peptide rAsKC11 was isolated by metal affinity chromatography on the Ni-NTA-agarose in the native condition from the soluble fraction of the cellular lysate after its ultrasound treatment or treatment using a French press.

The fusion protein was cleaved by treatment with cyanogen bromide. The target peptide, rAsKC11, was purified by RP-HPLC on a Jupiter C4 column (10 × 250 mm, Phenomenex, Torrance, CA, USA), equilibrated by 0.1% TFA, pH 2.2, in a gradient of ACN concentration (0–70%) for 70 min at 1.5 mL/min. 

### 4.3. Oocyte Preparation

All procedures for the use and handling of adult female *Xenopus laevis* frogs (CRB Xénopes, Rennes, France) were approved by the Animal Ethics Committee of the KU Leuven (Project No. P186/2019) following regulations of the European Union (EU) concerning the welfare of laboratory animals, as declared in Directive 2010/63/EU. Oocytes were isolated from ovarian tissue surgically removed during hypothermia and 0.1% (*m/v*) tricaine (Sigma-Aldrich Chemical, St. Louis, MO, USA)-induced anesthesia. After recovery from anesthesia, frogs were returned to their tanks in the Aquatic Facility of KU Leuven and were monitored daily.

The oocytes were enzymatically defolliculated by collagenase type IA (3 mg/mL) (Sigma-Aldrich Chemical, St. Louis, MO, USA) digestion at 16 ℃ on a rocker platform in a Ca^2+^-free ND96 solution. Isolated stage V-VI oocytes were then maintained in ND96 solution containing Theofylline and Gentamicin at 16 °C. The ND96 solution was composed of 96 mM NaCl, 2 mM MgCl_2_, 2 mM KCl, 5 mM HEPES, and 1.8 mM CaCl_2_, with a final pH of 7.5.

### 4.4. Electrophysiological Measurements

For the expression of potassium channels (hK_V_1.1, hK_V_1.2, hK_V_1.3, rK_V_1.4, rK_V_1.6, and mGIRK1/2, mGIRK2 alone, or IRK1) in *Xenopus* oocytes, the linearized plasmids were transcribed using the T3, T7, or SP6 mMESSAGEmMACHINE transcription kit (Ambion, Austin, TX, USA). Following the cRNA injection into oocytes and 1–4 days of incubation at 16 °C, electrophysiological experiments were conducted using the two-electrode voltage-clamp (TEVC) GeneClamp 500B (Axon Instruments, San Jose, CA, USA). Electrodes were fabricated from borosilicate glass tubes (1.14 mm outside diameter, 0.7 mm inside diameter) by a programmable microelectrode puller, PUL-1 (World Precision Instruments, Sarasota, FL, USA). Electrodes were filled with 3 M KCl and had tip resistances from 0.8 to 1.5 MΩ. Membrane currents from voltage-clamped oocytes were digitized using a Digidata 1550 low-noise data-acquisition system (Axon Instruments, San Jose, CA, USA) and a Dell PC running pCLAMP 10.4 software (Axon Instruments, San Jose, CA, USA).

For measuring the GIRK channels and IRK1, oocytes were placed in a 0.2 mL recording chamber continuously perfused with ND96 solution. After electrode impalement and clamping the membrane potential to −90 mV, the perfusion solution was changed to the HK solution composed of 96 mM KCl, 2 mM NaCl, 1 mM MgCl_2_, 1.8 mM CaCl_2_, 5 mM HEPES with a final pH of 7.5. The resulting increase in inward K^+^ current represents a basal K^+^ current that is following primarily G protein-independent GIRK channel activity. The direct application of AsKC11 to the bath was followed by the ceased perfusion of HK for 40–60 s and then AsKC11 was washed out by HK. This produced the reversible GIRK-dependent K^+^ current. Flow, through the perfusion system, was gravity-driven. K_V_1.x currents were evoked by 500 ms depolarizations to 0 mV followed by a 500 ms pulse to −50 mV, from a holding potential of −90 mV. All recordings were performed at room temperature (21 to 23 °C).

The spectrophotometric determination of AsKC11 concentration in the solution of the fraction A12 was conducted using the NanoDrop (ND-1000 UV/Vis, De Meern, the Netherlands). 

### 4.5. Data Analysis

All electrophysiological data are presented as means ± standard deviation (SD) of *n* ≥ 3 independent experiments unless otherwise indicated. All data were acquired using pClamp Clampex 10.4 (Axon Instruments, San Jose, CA, USA) and analyzed using pClamp Clampfit 10.4 (Axon Instruments, San Jose, CA, USA) and GraphPad Prism 8 software (GraphPad Software, Inc., San Diego, CA, USA). The statistical comparison between two experimental groups was performed by parametric unpaired *t*-test with Welch’s correction where *p* < 0.05 was considered significant.

The biomolecular reaction scheme which is compatible with a single exponential time course is referred to in a previous article [49], shown below:

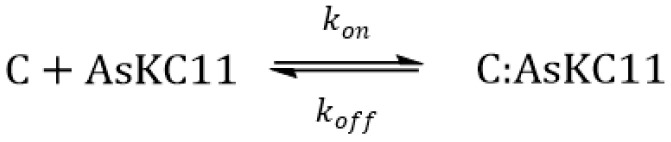

(1) $$k_{on}=\alpha \times \left[AsKC11\right]$$

(2) $$k_{off}=\beta$$

(3) $$\tau_{on}=\frac{1}{k_{on}+k_{off}}$$

(4) $$\tau_{off}=\frac{1}{k_{off}}$$

(5) $$k_{d}=\frac{\beta }{\alpha }$$

C = channel, C:AsKC11 = channel with bound AsKC11. *k_on_* and *k_off_* are the apparent first-order association and first-order disassociation rate constants, respectively. α and β are the second-and first-order rate constants of association and dissociation, respectively. *τ_on_* and *τ_off_* are the time constants for the approach to equilibrium upon wash-in and wash-out, respectively.

## Data Availability

The data presented in this study are available on request from the corresponding author. The data are not publicly available due to privacy.
